# Accessing local support online: Mothers' experiences of local Breastfeeding Support Facebook groups

**DOI:** 10.1111/mcn.13227

**Published:** 2021-06-01

**Authors:** Holly Morse, Amy Brown

**Affiliations:** ^1^ Department of Public Health, Policy and Social Sciences Swansea University Swansea UK; ^2^ Centre for Lactation, Infant Feeding and Translation Research (LIFT) Swansea University Swansea UK

## Abstract

The importance of support to breastfeeding success is well established, as are the difficulties many mothers face in accessing the support they need. With the majority of UK mothers now accessing social media for support, Breastfeeding Support Facebook (BSF) groups have increased exponentially. BSF groups vary in type (local or national/international) and in moderation—overseen by breastfeeding mothers and by midwives or trained lactation specialists. Some groups aimed at supporting mothers in a specific geographical area also have associated face‐to‐face groups, facilitated as either professional or peer support. Little is currently known about these specific local groups, their prevalence, impact or value to mothers. This paper examines mothers' experiences of using local BSF groups and why they value them as part of a larger study exploring the impact of midwife moderation on these groups. An online survey consisting of open and closed questions was completed by 2028 mothers. Findings identified that local BSF groups are widely used and highly valued for their connection with local face‐to‐face services and other mothers. They offer access to expertise and shared experience in a format mothers find convenient and timely, improving confidence and self‐efficacy. Local BSF groups enable the formation of support networks and development of breastfeeding knowledge that mothers credit with increased well‐being, motivation and breastfeeding duration. As such, they have the potential to add value to local face‐to‐face services and improve breastfeeding experiences and knowledge in communities. The findings have important implications to support the development of integrated online interventions to improve public health.

**Key messages**

- Local Breastfeeding Support Facebook (BSF) groups are common across the United Kingdom, often connected with face‐to‐face services and largely run by volunteers. They fill a gap in service provision.
- Mothers value the local aspect of the group for its convenience, signposting to other services, shared experience and developing real‐life connections with other breastfeeding mothers.
- Mothers join in pregnancy or the newborn period, often engaging with the group into toddlerhood, developing knowledge and social connections and sharing this with others.
- Local BSF groups form online communities that have the potential to improve services and breastfeeding duration in local areas.

## 1 BACKGROUND

Breastfeeding has not been the social norm in the United Kingdom for most of the last century (Jones, 2017). This means that new mothers no longer have a physical community of experienced and knowledgeable breastfeeding mothers able to offer them support (Brown, 2016). As a result, generations of parents have had to become increasingly reliant on health professionals and trained supporters for advice and expertise (Sinha et al., 2015).

Evidence shows that breastfeeding support should involve combinations of professional and peer delivery and should be targeted, predictable, accessible and delivered across a variety of clinical and community settings (McFadden et al., 2017; Sinha et al., 2015). Mothers need reassurance, opportunities for information sharing and discussion (Renfrew et al., 2012). A core aspect of support is through breastfeeding peer support groups. These groups enable mothers to access trained support, from health professionals, lactation specialists or peer supporters able to physically observe a breastfeed.

Research has established the positive practical and emotional impact of face‐to‐face peer support for mothers (McFadden et al., 2017). These groups offer reassurance when breastfeeding is going well and signposting where further input is needed, alongside social and emotional benefits through meeting other mothers and being in an environment where breastfeeding is normalised (Britton et al., 2007; Meadows, 2011).

However, mothers can face a number of logistical barriers to accessing the face‐to‐face support they need; they may be unwell after birth, have other children to care for or lack transport (Wagg et al., 2019). The COVID‐19 pandemic has exacerbated this, leaving new parents isolated and struggling to access the support they need (Brown & Shenker, 2020; Renfrew et al., 2020). Seeking breastfeeding support online offers a way to overcome these barriers (Bridges et al., 2018; Brown & Shenker, 2020). Facebook use peaks among women in pregnancy and is now commonly used by mothers seeking support in the transition to parenthood (Baker & Yang, 2018; Bartholomew et al., 2012). Utilising a format the majority of new UK mothers are familiar with, Breastfeeding Support Facebook (BSF) groups have become widespread, and research into their use and impact is growing (Black et al., 2020; Robinson et al., 2019; Skelton et al., 2018).

BSF groups form online communities, which provide informational and emotional support and opportunities for social learning (acquiring breastfeeding skills and knowledge from other members) (Skelton et al., 2020). However, support groups on social media can also present challenges for mothers, including identifying how to validate information and who is sharing it (Regan & Brown, 2019). Evidence‐based information sharing is key to the quality and efficacy of the support. Mothers express concern about the regulation of Facebook support groups and value moderation they can trust to address misinformation (Skelton et al., 2020).

As the use of Facebook groups for breastfeeding support becomes more widespread, we need to understand whether and how they can be combined with these services to best support mothers and families within their communities. Although research into online breastfeeding support has increased, there remains a gap in research about BSF groups specifically aimed at local populations of mothers. Little is known about their prevalence, links to local services and moderation. This study therefore aimed to explore how and why women find, use and value local BSF groups and who is providing them. Developing an understanding of the value of integrating high‐quality online support with local services will help inform practice and education, improving services for mothers and supporting funding cases for providers.

## 2 METHODOLOGY

### 2.1 Participants

Participants were mothers aged 18+ and breastfeeding at least one baby up to 24 months old. This range was chosen for analysis to reduce recall bias around reasons for joining the group and early experiences of feeding. All participants were currently a member of a local BSF group. This was defined as a Facebook group identified as offering breastfeeding support to mothers residing within any specific geographical area within the United Kingdom, rather than to national or international members. UK postcodes were provided to confirm residency. Exclusion criteria included age <18 years, inability to consent and inability to complete the questionnaire in English. Ethical approval was granted by a University Research Ethics Committee.

### 2.2 Questionnaire design

An exploratory online survey, consisting of open and closed questions, was designed to enable large‐scale, efficient data collection. Questions were devised from the literature on peer and online support, common support issues and reasons for breastfeeding cessation (Fox et al., 2015; McAndrew et al., 2012; Regan & Brown, 2019; Skelton et al., 2020).

The questionnaire (see supporting information) included items exploring:

- demographic background;
- current infant feeding mode, for example, breastfeeding exclusivity and formula use;
- format and function of the BSF group, for example, links to face‐to‐face support and who runs the group;
- reasons for joining, for example, breastfeeding problems and social reasons;
- experiences of receiving online support, for what and from whom; and
- perceptions of the value of belonging to an online BSF group.

The questionnaire was piloted in a named local BSF group prior to sharing and was completed by 12 mothers. Feedback from initial participants was positive on structure and content. No changes were required.

### 2.3 Procedure

Data were collected in January 2020. Participants were recruited to the study via a Facebook post containing brief details of the study and inclusion criteria and a link to the online questionnaire, hosted by Qualtrics. This was shared on Facebook and Twitter. Online recruitment alone was appropriate as participants were required to be current Facebook users. UK local BSF groups were identified via a Facebook search, with permission sought from group administrators for posting study information to the group. The post was also shared on the research team's social media pages with encouragement to share the link. The study advert received 449 shares over 14 days. If participants were interested in taking part, they clicked on the link where the participant information sheet and consent questions loaded. A short debrief was included at the end of the questionnaire with details of how to contact the research team or seek further support if needed.

### 2.4 Data analysis

Quantitative questionnaire data were analysed using SPSS v26. Descriptive data were analysed for frequencies. Cross‐tabulations were used to explore associations between baby age, group use and measures of support. Thematic analysis was conducted to explore patterns and connections within the qualitative data. After familiarisation with the data, initial codes were produced, identifying themes that were reviewed in relation to the coded extracts, defined and named. These were reviewed by a second researcher and discussed until agreement reached (Braun & Clarke, 2006). A reflexive journal was used to reflect on methodological decisions and the researcher's background in breastfeeding support and influences as a health professional. Confidence in the findings was developed via both prolonged engagement with and persistent observation of BSF groups prior to the study. Results were audited by the second researcher, providing feedback on the adequacy of data, development of findings and the interpretive perspective (Lincoln & Guba, 1985).

### 2.5 Ethical considerations

Ethical approval was granted by the Swansea University College of Human and Health Sciences Research Ethics Committee.

## 3 RESULTS

Two thousand and twenty‐eight mothers completed the questionnaire. Seventeen responses from participants residing outside the United Kingdom or referring to non‐local (national, international or issue‐specific) support groups were excluded from the analysis, leaving 2011 participants. Mean age of participants was 32.35 (SD: 4.551; range 19–47). Mean age of infants was 10.6 months (SD: 6.393; range 1–24) (Table 1).

**TABLE 1:** Sample distribution by demographic factors

Indicator	Group	*N*	%
Age	≤20	3	0.1
	21–25	9	0.4
	26–30	127	6.3
	31–35	547	27.0
	36–40	828	40.8
	≥41	420	20.7
Education	No formal	11	0.6
	GCSE	117	5.8
	A‐level	307	15.2
	Degree	883	43.9
	Postgraduate	692	34.4
Ethnicity	Asian or Asian British (Indian, Bangladeshi, Pakistani, other)	42	2.09
	Black or Black British	5	0.25
	Chinese	5	0.25
	Gypsy/traveller	1	0.05
	Irish	35	1.74
	Mixed or multiple	41	2.04
	Other	12	0.60
	White/White British	1872	93.0
Marital status	Married/civil partnership	1451	72.2
	Divorced	10	0.50
	Cohabiting	474	23.6
	Single	73	3.6
	Widowed	2	0.10
Employment	Full time	819	40.8
	Part time	828	41.2
	Not working	361	18.0

Abbreviation: GCSE, General Certificate of Secondary Education.

At the time of survey completion, 589 (29.5%) babies were receiving only breast milk (breastfeeding or pumped breast milk) and 1240 (62.1%) were weaned onto food but still receiving breast milk (Table 2). Of babies aged 0–6 months, 81 (8.4%) were receiving any formula, 504 (74.6%) were exclusively breastfeeding and 1.6% (*N* = 11) were receiving expressed breast milk only. Overall, 573 (97.1%) of the 590 babies aged over 6–12 months were continuing to receive some breast milk, and 40 (6.8%) babies were no longer breastfed.

**TABLE 2:** Sample distribution by baby's current method of feeding

Current feeding method	*N*	%
Breastfeeding only	574	28.7
Pumped breast milk	15	0.8
Formula milk	10	0.5
Combination feeding (breast milk/formula)	118	5.9
Solid food and breast milk	1240	62.1
Solid food and formula milk	40	2.0

### 3.1 Group prevalence and format

Participants were asked the location and name of the BSF group they belonged to. Participants belonged to 227 groups from across the United Kingdom. Although highly populated areas represented greater numbers of groups and responses, the spread demonstrated that local BSF groups are now widely available. When asked if it was important to them that other members were local, rather than from a wider UK or international area, 1255 (74.2%) agreed this was a valued feature.

Participants were asked a series of questions about the local BSF group format (e.g., who moderates it, i.e. takes responsibility for regulating posts and discussions, and any links to face‐to‐face breastfeeding support). Although 20.7% of mothers were unaware of who moderated the group, other options given included trained peer supporters (47.9%), lactation consultants (29.1%) and parents (19.9%). Some groups had mixed moderation across those categories. Overall, 1054 (67.0%) mothers indicated awareness of a linked local face‐to‐face breastfeeding support group, and 734 (69.8%) of those participants had attended it at least once. Together, lactation specialists and trained peer supporters were providing the majority of face‐to‐face support (60.9%) and online group moderation/support (77.0%).

### 3.2 Joining and using the group

Participants indicated how they had become aware of the BSF group (*agree*–*disagree* 5‐point Likert scale). The most common sources (*agree* and *strongly agree*) were through a recommendation from family or friends (43.9%), a Facebook search (43.8%), recommendations from midwives (31.2%) and leaflets (16.6%). Some participants noted more than one source. Overall, 659 (38.5%) mothers had been told about the online group at a face‐to‐face group, a positive impact of linked services on Facebook group promotion.

In terms of reasons for joining, participants were asked a series of questions surrounding their seeking online support, including when and why they joined the local BSF group and how often they used it. The range of joining was pregnancy to 18 months after birth. Of these, 1256 (61.9%) had joined the group to access breastfeeding support either in pregnancy or within 3 months of birth, and 289 (14.3%) had remained in the group since breastfeeding a previous child.

Participants rated a number of reasons for joining the BSF group (*agree*–*disagree* 5‐point Likert scale). *Agree* and *strongly agree* responses were aggregated (see Table 3). Although 41.7% joined because of experiencing a breastfeeding problem, 71.1% joined or remained in case of future problems. Table 2 shows the specific reasons for joining. Many participants joined for more than one reason, encompassing practical, emotional and social motivations. The most common reasons were for reassurance, access to shared experience and trained support.

**TABLE 3:** Reasons for joining the local Breastfeeding Support Facebook group

Motivation for joining	*Agree*/*strongly agree*	
	*N*	%
Reassurance about breastfeeding	1521	87.1
Reassurance about normal baby behaviour	1497	85.7
To share experiences	1366	78.1
To find like‐minded mothers	1344	77.0
In case of problems	1442	71.1
To access trained peer support	1223	69.8
To find a face‐to‐face group or support	890	50.9
To access support without attending a face‐to‐face group	522	50.9
Already having problems	721	41.7
Unable to attend a face‐to‐face group	392	22.5
To access midwifery support	251	14.4
No other support for breastfeeding	263	13.0

Participants were asked how often they used or visited the online group. Daily use or greater was most prevalent among mothers with babies aged under 3 months (55.4%) declining to 35% among mothers with babies aged 18–24 months. However, asked to rate frequency of BSF group use by reason, 77.9% mothers very often or often read posts without commenting, and it was more common to use the group to answer (29.8%) rather than ask questions (10.1%).

### 3.3 Support needs met by the group

Participants were asked to indicate whether they had personally received support from the BSF group on a range of common practical, social and emotional issues (*agree*–*disagree* 5‐point Likert scale). *Agree* and *strongly agree* responses were aggregated (Table 4). Support for pain (45.4%) and for breastfeeding older babies/toddlers (49.1%) were sought most, and private referrals (30.1%) sought least. Although responses were polarised on most issues, broadly equal numbers of participants agreed and disagreed that they had received support personally for each issue, suggesting that support for all of these was provided via the local BSF groups, available to those seeking it. The majority of this support was provided by other mothers (89.3%) and peer supporters (76.5%).

**TABLE 4:** Distribution of support by issue type

Issue	*Agree*/*strongly agree*	
	*N*	%
Pain	711	45.4
Lack of sleep	626	40.1
Safe bed‐sharing	604	38.7
Unsupportive friends/family	514	33.0
Feeding in public	665	42.6
Baby weight gain/loss	619	39.7
Increasing milk supply	570	36.6
Introducing formula	135	8.7
Mental/emotional health	606	38.8
Baby development	470	30.1
Weaning onto solids	557	35.7
Breastfeeding older babies/toddlers	765	49.1
Private service recommendations	468	30.1
NHS service/group/clinic recommendations	660	42.4

Abbreviation: NHS, National Health Service.

Mothers rated how often they had seen a list of common issues discussed on the BSF group (*very often*–*never* 5‐point Likert scale). *Often* and *very often* responses were aggregated. Table 5 shows the most commonly seen discussions centred around breastfeeding physiology, including positioning and attachment, frequency of feeding and increasing milk supply. Relationships and parenting styles were seen least but were still frequently discussed.

**TABLE 5:** Most frequently discussed topics

Topic	*Often*/*sometimes seen*	
	*N*	%
Frequency of feeding	1560	99.3
Complications	1557	99.1
Expressing breast milk	1550	98.8
Positioning/attachment	1547	98.5
Increasing milk supply	1546	98.4
Sleep	1538	97.9
Baby weight loss/gain	1537	98.4
Tongue tie	1535	97.8
Weaning	1511	96.1
Returning to work	1502	95.6
Bed‐sharing	1499	95.4
Baby development	1433	91.3
Formula feeding	1391	88.6
Social events	1381	87.9
Parenting styles	1317	83.8
Relationships	1272	80.9

### 3.4 Experiences of local BSF group membership

Mothers' experiences of using a local BSF group were explored via a series of positive and negative items (*agree*–*disagree* 5‐point Likert scale). *Agree* and *strongly agree* responses were aggregated. Table 6 shows the most popular experiences centred on the helpfulness of reading others experiences and learning more about breastfeeding physiology. Mothers also agreed group membership improved their knowledge and perceived the group to be a reliable source of information. Social and emotional experiences, such as connecting with other parents, receiving emotional support and taking enjoyment in offering support to others, were common. Negative experiences such as confidentiality or judgement were low but experienced by around a fifth of women.

**TABLE 6:** Experiences of group membership

Statement	*Agree*/*strongly agree*	
	*N*	%
Reading others' experiences is helpful	1546	98.5
Confidence in reliability of group information	1429	91.2
Increased knowledge of breastfeeding physiology/process	1424	90.9
Reassured by access to trained support on group	1313	84.0
Enjoy offering support to others	1222	78.2
Group gives emotional support	1191	76.1
Feel connected to other parents on group	971	62.2
Midwife contributions improve confidence in the advice	513	32.7
Aware/experienced judgemental or negative comments	308	19.6
Facebook confidentiality/privacy concerns	261	16.7
Access to midwifery support not available elsewhere	226	14.5

The key to membership and frequency of engagement with the BSF group was its perceived value to mothers as a source of support, based on a range of positive experiences. Participants were asked to further reflect, using open ended boxes, on whether and why they would recommend the group to others. Thematic analysis on these reasons for valuing the group identified four themes: convenience, expertise, community and self‐efficacy.

#### 3.4.1 Convenience

Two subthemes were identified under the concept of the BSF group being convenient for mothers. These include the value of being able to access information, advice and reassurance online at any time of day or night, receiving fast responses and extending access to local services.

##### Service

Mothers described BSF groups as filling a gap in local provision. This was particularly important for those who felt the loss of face‐to‐face services, describing local group provision as recognition of the importance of breastfeeding support to families.

Local support has almost disappeared. We with my eldest there was a breastfeeding cafe every single day of the week, twice on a Wednesday …. Now there is one a week. The helplines have closed. This Facebook group are run by the same people, but without funding, but people with a passion, and who care about children and mums. (Aged 37, baby 22 months)

Others described groups as extending their access to support and signposting other services.

The Facebook group provides a wealth of knowledge and signposting to specialist services I'd otherwise not known about or how to access. (Aged 33, baby 11 months)

##### Accessibility

The majority of mothers felt the value of the group in offering support and reassurance, accessible as and when it was needed most. This was frequently in the early hours of the morning, where support and solidarity could be accessed one‐handed during night feeds. Some mothers felt that the group supported them to develop wider knowledge through consistent online engagement, not just when a problem arose.

If it wasn't for being able to ask a question at any time (even 2am) I wouldn't be continuing to breastfeed. Without this type of group I would not have breastfed my first or second. Being able to get support and advice without having to physically go somewhere to meet people in person is exactly what I want. It also allowed me to understand wider issues and recognise if I was starting to develop them. (Aged 35, baby 6 months)

Mothers recognised the limited capacity and resources available to health professionals and saw access to local support online as bridging a gap between one‐to‐one care and physically attending a group.

Midwives and health visitors are limited in the amount of time they have to offer support on breastfeeding especially in the early days ‐ the Facebook group is incredibly supportive and accessible at 3am when you need it most and when it's not possible to get to one of the face to face groups. (Aged 26, babies 22 months and 1 month old)

#### 3.4.2 Expertise

Mothers highly valued access to trained and peer expertise via the BSF group. They described their confidence, self‐efficacy and learning being improved by reassurance from both trained supporters and health professionals alongside the solidarity and motivation provided by sharing experiences with other mothers. Two subthemes therefore arose—experience and training.

##### Experience

Trusted ‘real‐world’ lived experience, advice and reassurance from peers were highly valued. Mothers commonly reported that the BSF offered support that enabled and motivated continuation, particularly where it was lacking within their personal sources.

The Facebook group gave me support and suggestions that enabled me to continue, when my husband and family said I should stop. I only had support from the Facebook group, an amazing bunch of ladies. (Aged 30, baby 9 months)

The power of access to this variety of lived experience and solidarity was evident, including for those with professional training.

It is invaluable to have support from such a wide variety of mums on the group. There is almost always someone who has experienced the same problem and can offer advice. I am a midwife myself but had no training on oversupply and breastfeeding was very different experiencing it first‐hand as opposed to my midwifery training 5 years ago. (Aged 32, baby 17 months)

##### Training

Mothers described the value of having access to local trained support via the group that was not always available elsewhere.

Having an IBCLC qualified person so nearby and actively involved in the group is extremely valuable in my opinion. I have one friend who arranged a face to face consultation with her following feeding issues, something she couldn't have done if she hadn't been local to us. (Aged 36, baby 9 months)

The provision of an online service was seen to demonstrate a commitment to local breastfeeding mothers and passion for breastfeeding support.

Huge amount of support from highly trained, knowledgeable professionals. Much more helpful than information and help received from other services … Extremely fast responses. And people are clearly passionate about breastfeeding and supporting mothers to do so. One of the best resources available. (Aged 28, baby 6 months)

#### 3.4.3 Community

This theme described the value to mothers of the sense of local community built from shared experience and developing knowledge and expertise within the BSF group.

##### Village

Mothers highly valued the social capital developed by belonging to a community with a shared goal. They felt this had a positive impact on them and their breastfeeding. Mothers frequently referred to the BSF group and its members as a village or tribe with clear emotional connection.

It has been a life saver in both sanity and my breastfeeding journey …. It's the village of women who I know but have never met who have helped raise this mum and her babies. (Aged 31, baby 7 months)

The siting of the group within local services provided opportunities for social connections made online to become in person friendships with other breastfeeding mothers.

Sometimes all you need is a sounding board or reassurance that we're not in this alone, many other mums are experiencing the same thing and it's normal. Just to have a bit of guidance, and support, especially in the small hours when you're sleep deprived, hormonal and emotional, is wonderful. They're my tribe, we meet at least once a week in person. (Aged 42, baby 12 months)

##### Involvement

Mothers also valued the reciprocity of the BSF group format. They felt as their knowledge and experience grew they were able to support others online, giving back through involvement in the community.

It's essential to connect with other mums feeding. It's a real sense of community, I can also contribute and share my experience and knowledge to other new mums, and feel I can help and support others. (Aged 25, baby 4 months)

Many mothers had felt motivated to train as peer supporters themselves as a result of group experiences, demonstrating the way the support community continues to develop as a resource, particularly in areas that are otherwise underserved.

I started as a new mother feeling very supported by other Mums …. This has led me to train as a peer supporter myself, I've learned so much more and now provide support to others …. Living in a rural location this online resource is invaluable. (Aged 28, baby 5 months)

#### 3.4.4 Self‐efficacy

This theme describes how mothers value the role of the group in increasing their confidence in their ability to troubleshoot problems (for themselves and others), in feeling able and confident in sharing that knowledge within and outside of the group and to meet their own breastfeeding goals.

##### Learning

Mothers valued the role of the group in providing ongoing opportunities for learning. They felt group that membership resulted in greater knowledge acquisition through access to information and shared experience and that this increased confidence.

It is a wealth of information and important support. I am a paediatric doctor and I recommend it to new parents on an almost daily basis as it saved my breastfeeding journey, increased my knowledge exponentially. I value the experience of other mums hugely. (Aged 32, baby 8 months)

Mothers referred to the local BSF group as a ‘hive mind’, demonstrating the value of access to the collective and growing knowledge and experience within the online community.

Friendly, supportive advice from real mums with real babies, not textbook ones. Pretty much 24/7 advice/support available. Access to the hive mind and so many more like minded mums/mums going through the same issues you are having than you would normally have access to at a group. Found this hugely reassuring. (Aged 32, baby 16 months)

##### Success

The success of the group in offering support, resulting in improved experiences, longer breastfeeding duration and sharing within the geographical community was valued by mothers.

My own peers/family members have not followed my own experiences with the extended duration of breast feeding their own babies. I feel online I have found like‐minded women. Their knowledge and ability to display their hurdles as well as successes has helped to quietly empower my own experience. I will be sharing this group to any new mum. (Aged 31, baby 5 months)

Mothers felt that standard health professional support was unable to meet the evolving needs of breastfeeding mothers as effectively as those sharing in the experience. Solidarity was particularly important in facing challenges and increased motivation to meet and surpass their breastfeeding goals.

Really useful to have access to as much support as possible. When there are problems with breastfeeding e.g. baby won't feed with cold, baby biting, it can feel quite important to get quick support as it's a bit of a nightmare if you can't feed your baby or have to switch feeding type suddenly. My experience of midwife, HV and GP was that they could be hit and miss in terms of availability and knowledge. Having a community of mothers with direct experience, moderated by [lactation] experts very helpful. Also good for increasing my knowledge and not feeling isolated. (Aged 35, baby 19 months)

## 4 DISCUSSION

This study explored women's experiences of belonging to local BSF groups, specifically whether and how they are valued. The findings add to a growing body of research that shows the value of online breastfeeding support, specifically exploring how local groups for breastfeeding mothers living in a local area provide a valuable sense of support, information and community. The findings have important implications for the potential delivery of localised breastfeeding support through online formats.

Concurrent services across a combination of settings (hospital, home and community) are the most effective in optimising breastfeeding rates (Sinha et al., 2015), underlining the benefits of locating online support within geographical areas and health services. Mothers agreed that the local aspect of the BSF group was important, giving them access to information on local services and shared experience not available elsewhere. Mothers also valued the signposting and access to linked face‐to‐face groups to address challenges that required observation or in person support. The format also offers the opportunity for mothers to connect online and also meet up physically, either independently or at a face‐to‐face group. This enables ‘real‐life’ supportive relationships to develop between mothers, benefiting them and their babies socially and through shared breastfeeding knowledge. Local online support may also facilitate continuity of care between support providers and group members (McCarthy et al., 2017), a feature known to improve breastfeeding outcomes (Fox et al., 2015).

The findings highlight the prevalence of local BSF groups as a source of support now accessed by a large number of breastfeeding mothers across the United Kingdom. Responses were received from mothers spread across all four nations with babies ranging from 0 to 24 months old. Group use was most frequent among those with the youngest babies, with many reporting at least daily visits. Around a quarter of participants had joined the group pre‐emptively during pregnancy meaning they knew where to find support when needed when their baby was born. We know this is a critical time to receive support; breastfeeding rates drop rapidly in the days and weeks after birth (Lancet, 2016).

Mothers used the group not only to seek support for practical aspects of breastfeeding like positioning at the breast, frequency of feeding and expressing milk but also to access support on wider issues like safe bed‐sharing and breastfeeding in public. Participants talked about the group ‘ethos’ around these wider issues as helping them to continue to breastfeed by normalising these approaches. We know this feeling or normalisation is important in helping women breastfeed for longer (Fox et al., 2015). Almost a decade ago, research started to highlight how social media platforms such as Facebook can provide a platform for accessing new networks, supporting successful adjustment to parenthood (Bartholomew et al., 2012).

Mothers talked about how beneficial the group was for not only practical information but also wider community connections and support. BSF groups provide a stepping stone to accessing face‐to‐face support (Regan & Brown, 2019), and we found local groups anchor this within a physical community, enhancing the shared experience and opportunities for social networking. Online communities thrive on reciprocity and interaction (Coulson & Smedley, 2015; Skelton et al., 2020), and some mothers clearly benefited from engagement, both being able to ask and respond to others. Notably, mothers did not appear to necessarily need to interact to feel a sense of belonging, support and connection; reading posts without commenting was actually the most common type of use.

We found that this connection was important for mothers who did not have a supportive family or community around them in day‐to‐day life. A lack of embodied experience of friends and family breastfeeding influences whether a mother decides to stop breastfeeding herself (Fox et al., 2015). Mothers often described a lack of breastfeeding experience and knowledge in their family and social network, even where they were supportive. This is a common experience, where despite their intention/desire to continue breastfeeding, mothers are faced with a dearth of knowledge and experience among their family and wider social network, for whom converting to formula feeding is often the solution to every problem (Brown, 2015). As in other studies (Black et al., 2020; Skelton et al., 2020), mothers felt that the BSF group was able to counter this lack of support, experience or misinformation in their existing networks.

Mothers credited membership of the group with longer breastfeeding duration, with some reporting they had continued beyond their initial goals due to the support and connection received in the group. Others expressed pride at having overcome significant difficulties to continue. Although this is a self‐selecting sample, likely of more motivated individuals, these beliefs, alongside the high proportion of breastfeeding beyond 6 months among BSF group members, suggest that group membership supports mothers in reaching their breastfeeding goals. McFadden et al. (2017) found that support interventions with a face‐to‐face component are most effective, and many local BSF groups were providing this during data collection, which was prior to the outbreak of COVID‐19. Mothers described access to linked support as useful or reassuring, but notably, others felt the online group alone met all their needs. Online support has become vital where face‐to‐face support has been withdrawn during the COVID‐19 pandemic (Brown & Shenker, 2020), and future reviews may identify changes to patterns of BSF use and its impact.

Longer term membership of the group is also an important element to highlight. Overall, 65.5% of our sample was breastfeeding babies older than 6 months old. This highlights the significant value of the group; although mothers remained for support and queries relating to older babies, they also remained for the connection with the community beyond the need for advice. This led to a growing wealth of community knowledge and advice being built. Mothers of older babies often offered guidance and emotional support for those with younger infants, passing on information and ideas that they had been given when their own babies were younger. This human capital and tacit knowledge of both how to support new mothers practically and emotionally provide rich depth to the groups (Pyrko et al., 2017).

These mothers also modelled the concept of longer term breastfeeding, providing much needed normalisation of this within communities where breastfeeding rates were particularly low. As social beings, we are affected by the images that we see around us, yet most imagery of breastfeeding focuses on much younger infants (Dowling & Brown, 2013). Given only a third of mothers in the United Kingdom breastfeed past their infant turning 6 months old, and far fewer into the second year and beyond (Victora et al., 2016), these mothers may be the only reference many mothers of younger infants have of breastfeeding past the early months.

Delivery of these groups is an important aspect to consider. The majority of local BSF support is being provided on a volunteer basis by other parents, peer supporters and lactation consultants. This raises a number of important issues. First, it highlights the issue of a considerable amount of breastfeeding support being offered by volunteers. The groups were reliant on mothers bringing lived experience and acquired knowledge acquisition to the group, sometimes bolstered by formal training. Although peer support is a common avenue of emotional support and feeling of community across a range of health issues, breastfeeding support is fairly unique in its reliance on peers and volunteers to often give practical information about how to make breastfeeding work (Grant et al., 2018; Regan & Brown, 2019). This feeds into a wider issue of the underfunding and undervaluing of breastfeeding and its significant impact upon public health and the economy (UNICEF, 2017).

There are also issues relating to the regulation and moderation of the information given within these groups. The role of moderator is key to the function of online support communities, with moderators addressing misinformation and facilitating respectful online discussion (Grimmelmann, 2015). Some mothers said that they experienced difficulties with recognising who was moderating or offering trained support and struggled to trust and verify sources. Where moderation is lacking or divisive, mothers can experience polarised debate, negativity and judgement, driving concerns about lack of regulation (Regan & Brown, 2019). Knowing who was providing them with trained support led to mothers visiting the group more often and believing it to be reliable. This has important implications for developing local BSF groups as an effective breastfeeding support intervention, and further analysis will focus on this aspect.

The research does have limitations. Participants were self‐selecting and more likely to represent the most motivated to take part. Additionally, participants were all current members of a BSF, and it is likely that those who had ambivalent or negative experiences leading to them leaving such a group are absent from our data. Further research is needed to understand these experiences and group attrition rates. Moreover, as is often the case in health research, mothers were older and with a higher rate of education than the population average, although this would be skewed by older women with a higher level of education breastfeeding for longer and thus being part of such groups (McAndrew et al., 2012). Findings should be treated with caution but do provide insight into the experiences of women using these groups for support.

Linked to this, our sample was predominantly from White or White British backgrounds (93%). This may be due to many self‐selecting health studies underrecruiting those from ethnic minority backgrounds. However, it may also be that women from ethnic minority backgrounds are less likely to join breastfeeding peer support groups, despite having higher breastfeeding rates in the United Kingdom, than White women (McAndrew et al., 2012). Ingram et al. (2008) found that there were also variations in how women from different ethnic minority groups preferred to receive breastfeeding support—some communities preferred groups aimed at their specific ethnic group, and others felt the group should be open to all. Those that seek support online struggle to find local groups that reflect and share their experiences, with Black British mothers reporting joining American BSF groups solely for Black women to feel part of a relatable breastfeeding community (CIBII UK, 2018).

Evidence highlights barriers to participation including a predominance of White peer and professional leaders of these groups, group content failing to take into account issues of diversity relevant to ethnic minority communities and a lack of cultural sensitivity (La Leche League, 2020).

Although BSF groups also exist offering support grouped by other common factors (e.g., ethnicity, age and profession), recruitment for this study focused specifically on local BSF groups. The prevalence and membership profile of BSF groups of other types (non‐local) requires further research to establish whether engagement in these by those typically less likely to initiate breastfeeding is greater than within the local groups explored by this study. Wider understanding will inform whether local BSF group provision should include minority groups or offer specialised services.

Limitations aside, the findings are an important exploration of a novel area: the value of local BSF groups and their potential to support breastfeeding within communities.

Mothers described the ways in which they use and perceive local BSF groups, highlighting them as a now vital and valued source of support. Although data were collected prior to the COVID‐19 pandemic, the importance of online support is highlighted by the reduced face‐to‐face services and uncertainty surrounding COVID‐19, and it is likely that results would be affected by this context. Although findings are preliminary, they highlight the potential of the local BSF group format to improve public health outcomes and support maternity services to fulfil strategic goals, providing a foundation on which to establish an evidence base for the provision of local BSF groups within health services.

## CONFLICTS OF INTEREST

The authors declare that they have no conflicts of interest.

## CONTRIBUTIONS

HM was responsible for the study design, data collection, data analysis, draft writing and critical revisions. AB was responsible for the study design, data analysis support, draft writing support and critical revisions.

## Supporting information



**Data S1.** Supporting Information

## Data Availability

The data that support the findings of this study are available from the corresponding author upon reasonable request.
