# Supplementary material for: Accessing local support online: Mothers' experiences of local Breastfeeding Support Facebook groups
Source: Matern Child Nutr. 2021 Jun 1;17(4):e13227. doi: 10.1111/mcn.13227 (PMC8476430; doi:10.1111/mcn.13227)
Supplement: Supplementary file 1 — Data S1. Supporting Information [file MCN-17-e13227-s001.docx]

Q0.2 If you would like to take part in the questionnaire please read through the following statements.

- I have read and understood the information above.
- I am over 18 years of age.
- I have a baby aged 0 – 24 months.
- I am currently a member of a local Facebook group that offers breastfeeding support.
- I consent to taking part in this study.

If you can answer yes to all the above answers then click next to progress to the next page, otherwise thank you for your time.

Q1 What is your sex?

________________________________________________________________

Q2 How old are you?

________________________________________________________________

Q3 What is your highest level of education?

- No formal qualifications
- GCSE or equivalent
- A-Level or equivalent
- Degree or equivalent
- Postgraduate or equivalent

Q4 What is your marital status?

- Married/civil partnership
- Divorced
- Cohabiting
- Single
- Widowed

Q5 What are the first three letters of your postcode?

________________________________________________________________

Q6 Are you currently employed?

- Yes full time
- Yes part time
- No

Q7 What is your ethnicity?

- White/White British
- Gypsy/Traveller
- Irish
- Asian or Asian British: Pakistani
- Asian or Asian British: Bangladeshi
- Asian or Asian British: Indian
- Asian or Asian British: Chinese
- Asian or Asian British: Other
- Black or Black British
- Mixed or multiple
- Other

Q8 How old is your baby (or babies if twins or more)

________________________________________________________________

Q9 How are you currently feeding your baby?

- Just breastfeeding
- Just pumped breastmilk
- Just formula milk
- Combination/mixed feeding (formula and breastmilk)
- Breastmilk and solid food
- Formula and solid food

End of Block: Default Question Block

Start of Block: Block 1

Q11 Since deciding to breastfeed I have been well supported (in person rather than online) by:

|  | Strongly Agree | Agree | Somewhat agree | Neither agree nor disagree | Somewhat disagree | Not applicable |
| --- | --- | --- | --- | --- | --- | --- |
| My Family |  |  |  |  |  |  |
| My partner |  |  |  |  |  |  |
| My partner's family |  |  |  |  |  |  |
| Friends |  |  |  |  |  |  |
| Midwives |  |  |  |  |  |  |
| Health visitors |  |  |  |  |  |  |

Q13 *For the purpose of this questionnaire we want to know about how you use a local Breastfeeding Support Facebook group in your area. The type of group we are interested in is one that is specifically local to you. For example it might have the name of your town or area in the group name, as against a group that is accessed from people around the country / world and is a more general breastfeeding interest group.*

Q14 What is the full name of the local Facebook group you belong to which you joined for breastfeeding support? (Please enter the most used if you use more than one)

________________________________________________________________

________________________________________________________________

________________________________________________________________

________________________________________________________________

________________________________________________________________

Q15 How old was your baby when you joined the group?

________________________________________________________________

Q16 If you were pregnant or joined in a previous pregnancy/whilst breastfeeding previously please state:

________________________________________________________________

________________________________________________________________

________________________________________________________________

________________________________________________________________

________________________________________________________________

Q17 Why did you join the Breastfeeding Facebook group?

|  | Strongly agree | Agree | Neither agree nor disagree | Disagree | Strongly disagree | Not applicable |
| --- | --- | --- | --- | --- | --- | --- |
| In case I experienced breastfeeding problems |  |  |  |  |  |  |
| I was already having breastfeeding problems |  |  |  |  |  |  |
| For reassurance about how breastfeeding was going |  |  |  |  |  |  |
| For reassurance about normal baby behaviour |  |  |  |  |  |  |
| To share experiences of breastfeeding with other mothers |  |  |  |  |  |  |
| To find like minded mothers |  |  |  |  |  |  |
| I was looking to find face to face support or a group to attend |  |  |  |  |  |  |
| I wanted support but didn’t want to go to a face to face group |  |  |  |  |  |  |
| I wanted support but couldn’t get to a face to face group |  |  |  |  |  |  |
| I had no other support for breastfeeding |  |  |  |  |  |  |
| For support from midwives on the group |  |  |  |  |  |  |
| For support from trained peer supporters on the group |  |  |  |  |  |  |

End of Block: Block 1

Start of Block: Block 2

Q18 How did you become aware of the Breastfeeding Facebook Group?

|  | Strongly agree | Agree | Neither agree nor disagree | Disagree | Strongly disagree | Not applicable |
| --- | --- | --- | --- | --- | --- | --- |
| My community midwife recommended it |  |  |  |  |  |  |
| A hospital midwife recommended it |  |  |  |  |  |  |
| Saw information leaflet or poster in hospital or clinic |  |  |  |  |  |  |
| Saw information leaflet or poster in community e.g. supermarket, library |  |  |  |  |  |  |
| I did a Facebook search |  |  |  |  |  |  |
| I did a google search for support groups |  |  |  |  |  |  |
| I attended the Face to Face group associated with the online group first |  |  |  |  |  |  |
| I saw the group details shared on Facebook/social media |  |  |  |  |  |  |
| Friends/family members recommended it |  |  |  |  |  |  |

Q19 Is the online Breastfeeding Facebook group associated with a Face to Face support group?

- Yes
- No
- Maybe

Display This Question:

If Is the online Breastfeeding Facebook group associated with a Face to Face support group? = Yes

Q20 Have you attended the Face to Face support group?

- Yes
- No

Display This Question:

If Have you attended the Face to Face support group? = No

Q21 Why have you not attended the Face to Face group?

________________________________________________________________

________________________________________________________________

________________________________________________________________

________________________________________________________________

________________________________________________________________

Display This Question:

If Is the online Breastfeeding Facebook group associated with a Face to Face support group? = Yes

Q22 Who runs/offers support at the Face to Face support group? (Please tick all that apply)

- Parents
- Midwives
- Maternity Support Worker
- Health Visitors
- Lactation specialist e.g. IBCLC, LLL, ABM
- Trained peer supporters
- Health Visitors
- Unsure

Q23 Who runs/moderates the Facebook Group? (Please tick all that apply)

- Parents
- Midwives
- Maternity Support Worker
- Health Visitors
- Lactation specialist e.g. IBCLC, LLL, ABM
- Trained peer supporters
- Health Visitors
- Unsure

Q25 How do you know who runs/moderates the Facebook Group? (Please tick all that apply)

- It is written in the group description
- They introduced themselves on the group
- The person who recommended the group told me
- I saw details on a leaflet or shared post
- I’ve worked it out myself reading group posts
- I’ve met them at a face to face group
- I’ve had care from them outside the group (e.g. as a community or hospital midwife)
- I don’t know who runs it

Q26 Are you aware of any group members who are who are local midwives commenting in a professional capacity?

- Yes
- No
- Unsure

Q27 How important to you is it that parents using the Breastfeeding Facebook Group are from your local area?

- Very important
- Moderately important
- Slightly important
- Not at all important
- Unsure

Q28 If you have had or seen support from Midwife members/moderators in the Breastfeeding Facebook Group how useful did you find this?

- Very useful
- Useful
- Not useful
- Not applicable

Q29 If the Breastfeeding Facebook Group does not have Midwife members/moderators do you feel having some would be valuable for the group?

- Yes
- Maybe
- No
- Not applicable

End of Block: Block 2

Start of Block: Block 3

Q30 How often do you use or visit the Breastfeeding Facebook group?

- Several times a day
- At least once a day
- A few times a week
- Once a fortnight
- Rarely

Q31 How often do you use the Breastfeeding Facebook group for the reasons below?

|  | Often | Sometimes | Rarely | Never |
| --- | --- | --- | --- | --- |
| To ask a breastfeeding question |  |  |  |  |
| To ask a baby related or parenting question e.g. sleep, weaning |  |  |  |  |
| To answer a breastfeeding question |  |  |  |  |
| To answer a baby related or parenting question e.g. sleep, weaning |  |  |  |  |
| To share links (e.g. events or information relevant to the group) |  |  |  |  |
| To read discussions without commenting |  |  |  |  |

Q32 Have you seen any of the following topics discussed on the group?

|  | Often | Sometimes | Rarely | Never | Unsure |
| --- | --- | --- | --- | --- | --- |
| Positioning and attachment |  |  |  |  |  |
| Frequency of feeding |  |  |  |  |  |
| Baby weight loss/gain |  |  |  |  |  |
| Increasing milk supply |  |  |  |  |  |
| Sleep |  |  |  |  |  |
| Bed sharing |  |  |  |  |  |
| Expressing breastmilk |  |  |  |  |  |
| Formula or bottle feeding |  |  |  |  |  |
| Tongue tie |  |  |  |  |  |
| Complications e.g. mastitis, thrush |  |  |  |  |  |
| Parenting styles |  |  |  |  |  |
| Social events/meet-ups |  |  |  |  |  |
| Baby development |  |  |  |  |  |
| Returning to work |  |  |  |  |  |
| Weaning |  |  |  |  |  |
| Relationships e.g. partners or family |  |  |  |  |  |

Q33 Have you received support from the Breastfeeding Facebook group with any of the issues below?

|  | Strongly agree | Agree | Neither agree nor disagree | Disagree | Strongly disagree | Not applicable |
| --- | --- | --- | --- | --- | --- | --- |
| Pain |  |  |  |  |  |  |
| Lack of sleep |  |  |  |  |  |  |
| How to safely bedshare |  |  |  |  |  |  |
| Dealing with unsupportive friends/family |  |  |  |  |  |  |
| Concerns about feeding in public |  |  |  |  |  |  |
| Concerns about baby weight gain/loss |  |  |  |  |  |  |
| Increasing milk supply |  |  |  |  |  |  |
| Introducing formula |  |  |  |  |  |  |
| Mental or emotional health |  |  |  |  |  |  |
| Questions about baby development |  |  |  |  |  |  |
| Weaning onto solids |  |  |  |  |  |  |
| Breastfeeding older babies/toddlers |  |  |  |  |  |  |
| Recommendations for private tongue tie services and/or osteopathy |  |  |  |  |  |  |
| Recommendations for NHS services, groups or clinics |  |  |  |  |  |  |

Q34 Or support for any other issue that was important to you? Please give details:

________________________________________________________________

________________________________________________________________

________________________________________________________________

________________________________________________________________

________________________________________________________________

Q35 Did you feel supported on these issues by:

|  | Very often | Often | Sometimes | Rarely | Never | Unsure | Not applicable |
| --- | --- | --- | --- | --- | --- | --- | --- |
| Midwives |  |  |  |  |  |  |  |
| Trained peer supporters |  |  |  |  |  |  |  |
| Other parents |  |  |  |  |  |  |  |
| Lactation specialists e.g. IBCLC, LLL, ABM |  |  |  |  |  |  |  |

Q36 Do you agree with the following statements about your experiences of using the group?

|  | Strongly agree | Agree | Neither agree nor disagree | Disagree | Strongly disagree | Not applicable |
| --- | --- | --- | --- | --- | --- | --- |
| I find reading other mothers’ breastfeeding experiences helpful |  |  |  |  |  |  |
| I feel confident that the information on the group is reliable |  |  |  |  |  |  |
| I feel more confident taking advice if midwives add to the discussion |  |  |  |  |  |  |
| I get emotional support from the group |  |  |  |  |  |  |
| I have seen judgemental or negative comments |  |  |  |  |  |  |
| My knowledge of how breastfeeding works/what is normal has grown since joining the group |  |  |  |  |  |  |
| I am reassured by having access to trained support via the group |  |  |  |  |  |  |
| I enjoy supporting other parents by sharing my experiences and knowledge |  |  |  |  |  |  |
| I feel connected to other parents on the group |  |  |  |  |  |  |
| I have concerns about privacy and confidentiality on Facebook |  |  |  |  |  |  |
| I feel I have access to midwifery support not available elsewhere |  |  |  |  |  |  |

Q37 Do you think there are any negatives to belonging the Breastfeeding Support Facebook group?

- Yes
- No
- Maybe

Display This Question:

If Do you think there are any negatives to belonging the Breastfeeding Support Facebook group? = Yes

Q38 What do you think the negatives to belonging to the group are?

________________________________________________________________

________________________________________________________________

________________________________________________________________

________________________________________________________________

________________________________________________________________

Q39 Would you recommend this group to other parents?

- Yes
- Maybe
- No

Q40 What are your reasons for recommending or not recommending this group?

________________________________________________________________

________________________________________________________________

________________________________________________________________

________________________________________________________________

________________________________________________________________

End of Block: Block 3
